# Using a three-dimensional computational fluid dynamics model and an agglomerate model to investigate the effect of varying agglomerate parameters and output voltages on proton exchange membrane fuel cell performance

**DOI:** 10.1016/j.heliyon.2024.e32277

**Published:** 2024-05-31

**Authors:** Abdelaziz Samris, Hamid Mounir

**Affiliations:** EMISys Research Team, Engineering 3S Research Center, Mohammadia School of Engineers, Mohammed V University in Rabat, Rabat, Morocco

**Keywords:** *PEM fuel cell*, *Agglomerate model*, *Agglomerate parameters*, *Platinum-oxide coverage*, *CFD methods*, *Output voltages*, *Cell performances*

## Abstract

Modeling of proton exchange membrane (PEM) fuel cells is attracting more attention as fuel cell technology continues to develop. In this study, we considered a hybrid model that combines an agglomerate model based on the agglomeration of catalyst particles and the coverage-dependent kinetic equation of platinum oxide for ORR, and another 3D numerical model of a PEM fuel cell based on computational fluid dynamics (CFD). The obtained results from our developed models were validated with experimental results from literature. In fact, we investigated the effects of changing the agglomerate radius $\left(R_{agg}\right)$, the ionomer volume fraction within the agglomerate $\left(Y_{i,agg}\right)\text{,}$ the effective agglomerate surface area $\left(A_{i,agg}\right)$, the distribution of the gases and the temperature on the cell performances.

The results revealed that the cell performances are strongly influenced by changing $R_{agg}$ and $Y_{i,agg}$ for medium and high current densities: The activation loss increases with increasing $R_{agg}$ and decreasing $Y_{i,agg}$. Also, $A_{i,agg}$ increases with decreasing $R_{agg}$ and increasing $Y_{i,agg}$. In addition, the PEM fuel cell's power output is significantly enhanced when $R_{agg}$ is decreased and $Y_{i,agg}$ is increased, the optimal power being obtained for values of $R_{agg}=100nm$ and $Y_{i,agg}$ = 0.6. The numerical results also showed that decreasing the output voltage from 0.95V to 0.35V can accelerate the electrochemical reaction process.

## Highlights

• A hybrid model that combines an Agglomerate model and 3D-CFD model of PEM fuel cell was developed and validated with experimental result.
• The PEM Cell performances are strongly affected by the variation of agglomerate parameters ( $R_{agg}$ and $Y_{i,agg}$ at medium and high current densities.
• The process of electrochemical reaction is affected by changing the output voltage.
• Cell performances are significantly enhanced by decreasing $R_{agg}$ and increasing $Y_{i,agg}$.

## 1 Introduction

Technologies for generating green energy have become increasingly crucial due to the desire to reduce fossil fuel consumption and environmental pollution. One such technology for generating renewable and clean energy is fuel cell systems [1], and one of the best technologies for transforming energy in such systems is the proton-exchange membrane fuel cell (PEMFC), which has attracted considerable attention in recent times. Fuel cells can have both stationary and mobile applications thanks to the ability to vary their power output. For example, they could provide electricity needed to run smartphones and other mobile devices, or they could be used to power large systems, such as cars, buses, and power generators [2]. Unfortunately, issues with the durability, cost, and stability of fuel cell systems have limited the development and commercialization of the technology, especially for PEM fuel cell systems. Indeed, any commercialization will need to be based on lowering the cost by reducing the platinum loading, as well as improving cell performance and durability [3,4]. Several approaches have been attempted in this context for optimizing operating conditions [5], with various effects having been studied [6,7]. In particular, significant effort has been directed at reducing the platinum load in the catalyst layers (CLs) without compromising cell performance [8,9].

A PEMFC comprises various parts, including the bipolar plates (BPs), the gas diffusion layers (GDLs), the microporous layers (MPLs), the catalyst layers (CLs), and the membrane (PEM). The CLs are where the electrochemical reaction occurs, so they are a key component of a PEMFC, and this is where the electrochemical reaction rates are controlled. It generally comprises a void region sandwiched between ionomer- and carbon-supported Pt (Pt/C). On the anode side, hydrogen is oxidized to form two protons and two electrons (HOR), while on the cathode side, the protons and electrons are combined with oxygen to form water (ORR). Between these two reactions, the ORR is relatively much slower, so it is the most important determining factor of performance in a PEMFC. New methods are therefore urgently required to investigate ways to improve performance, especially for the cathode side of the CL where ORR is usually slow and ineffective [10]. Thus, to accurately and realistically predict the behavior of a PEM fuel cell, it has become vital for researchers to develop better modeling approaches for the cathode side of the CL. In general, three types of models have been proposed for characterizing and describing the cathode side of the CL, namely the interface model [11,12], the homogeneous model [13,14], and the agglomerate model [15,16].

Various numerical studies of the cathode side of the CL in a PEMFC with various characteristics have been presented in the literature [17]. Moein-Jahromi et al. [18] investigated the impact of changing several CL structural and cell-operation factors, while Jung et al. [19] simulated and analyzed different configurations of the carbon-supported Pt (Pt/C), ionomer, and void region in a CL. Approaches for CL modeling have become increasingly more sophisticated over the years, and these have been thoroughly discussed in one paper [20]. Of the three models mentioned above, the interface model is the easiest to use because it describes all CL activity as boundary conditions that are imposed at the proton-exchange membrane's interface with the gas diffusion layer. The homogeneous model, meanwhile, approximates the CL as a permeable structure of platinum, carbon, electrolyte and gas pores, and it is a more advanced approach. Nevertheless, the most complete model so far is the agglomerate model [21,22], which depicts CL activity as a multiple-scale issue.

In the agglomerate model, the CL comprises gas pores, agglomerates of Pt/C particles, and electrolyte films. These agglomerates are conceptualized as ionomer spheres filled with various Pt/C combinations [23]. Compared to models with a thin film and discrete catalyst volume, these agglomerate models are more conceptually deep and precise [24]. Siegel et al. [25] used this model to more precisely forecast the polarization curve. In order to generate non-uniform agglomerate cathode CLs, Song et al. [26] and Yin et al. [27] quantitatively examined several PEM fuel cells by varying the agglomerate radius, ionomer volume percentage, and porosity within the agglomerate. More recently, some scientists have suggested altering the classical agglomerate model by including numerical solutions to the micro-scale issue into the device-level simulation, such as in the work of Kamarajugadda and Mazumder [28].

Recently, computational fluid dynamics (CFD) models have proven to be invaluable tools for modeling various processes [29,30]. CFD can offer valuable insights into the development of PEM fuel cell processes [[31], [32], [33]]], allowing for the investigation of both transport and electrochemical phenomena. According to a literature review, it is evident that various numerical simulation approaches, including 1D, 2D and 3D models, have been utilized for studying the physical and chemical processes within PEM fuel cells, especially, 3D modeling of fuel cells presents challenges and requires certain assumptions. However, due to the intricate structures and processes within these small-scale fuel cells, traditional models often face limitations in both computational accuracy and efficiency. On the other hand, some researchers have employed simplified approaches, such as 1D modeling, to elucidate heat and mass transfer dynamics and electrochemical reactions within fuel cells [34,35]. Ferreira et al. [36] devised a hybrid 1D+3D numerical model to investigate the distribution of current density and the transport of liquid water within the serpentine channel of a PEM fuel cell. In this method, the 1D model was employed to compute parameters such as current density, liquid water formation, and oxygen consumption within the cell. Meanwhile, Das et al. [35] utilized a 3D Volume Fluid (VOF) model to investigate the transport and accumulation of water within the cathode gas channel of a PEM fuel cell. In the same context, this work proposes an approach that integrates the computational fluid dynamics (CFD) method with a 3D model. The novelty of this approach lies in its ability to ensure and analyze in detail the impact of output voltage on fuel cell performance and the evolution of mass fraction of gases and other variable along fuel cell.

This main focus of this study was on developing an agglomerate model of a cathode CL based on agglomerating catalyst particles and the Pt-oxide-coverage-dependent kinetics equation for ORR, as well as a three-dimensional (3D) numerical model of a PEM fuel cell using computational fluid dynamics (CFD) methods and the ANSYS FLUENT software. In this proposed agglomerate model, the geometry of the computational field is introduced, such that the governing equations at any point in the channels, GDLs, MPLs, and CLs in three dimensions are described, while the total mass and momentum conservation equation, the gas species conservation equation, and the energy conservation equation were also developed. In addition, the effects of varying the agglomerate model parameters, such as the agglomerate radius ($R_{agg}$) and the volume fraction of the ionomer ($Y_{i,agg}$) within the agglomerate, on the PEM fuel cell's performance were also investigated. We also developed a 3D numerical model of a PEM fuel cell based on the CFD simulation is presented. The effects on concentration, mass fraction of the gases, and temperature distribution were also investigated.

## 2 Model development

For this section, an analytical model of a mono-channel PEMFC was developed that took into account all the PEMFC's parts, such as the BPs, the GDLs, the CLs, and the PEM. The reactant gases, namely oxygen and hydrogen, were delivered into the gas flow channel, so they could diffuse into the catalyst sites of the anode and cathode sides through the porous GDL. Protons and electrons from the HOR at the anode were transferred to the cathode through the membrane and ultimately to an external circuit. When these protons and electrons combine with the oxygen present at the cathode's active catalyst, an ORR will eventually occur and produce water. The reaction here at the cathode determines much of the cell's performance, since the ORR at the cathode is significantly slower than the HOR at the anode [37,38].

### 2.1 Agglomerate CL Sub-model

In the proposed model, the CL is regarded as a matrix of spherical agglomerates, much like those shown in Fig. 2a, and we presume that these agglomerates may contain both bare carbon particles and Pt/C particles. Fig. 2b gives the schematic of a single agglomerate based on a real CL. There is a thin ionomer film covering each of these agglomerates.

**Fig. 1:**
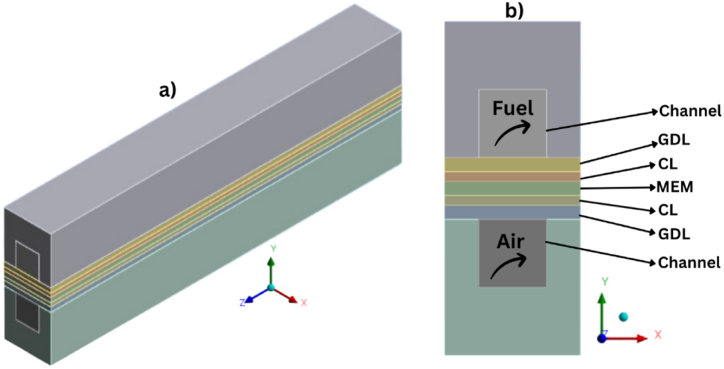
Schematic of the computation geometry for a single-channel PEMFC in (**a**) 3D and (**b**) 2D.

**Fig. 2:**
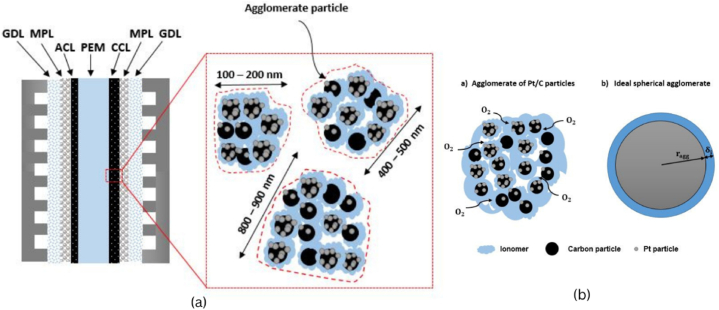
(**a**) Schematic of a PEMFC's geometry and cathode CL, as well as (**b**) an agglomerate of Pt/C particles together with an idealized representation of an agglomerate.

The effectiveness factor approach [39] and the spherical agglomerate model were employed to explain the electrochemical phenomena of interest and calculate the volumetric current density in the catalyst layer. The effectiveness factor is the ratio of the reaction rate within an agglomerate in relation to the hypothetical reaction rate under an infinitely simple transport.

### 2.2 CL composition and volume fraction

The CL comprises Pt/C particles, an ionomer phase, and a pore phase. The volume fraction of each element is determined based on several parameters, such as the thickness of the CL ($\gamma_{CL}$), the density of the carbon ($\rho_{C}$), the density of the platinum ($\rho_{Pt}$), the platinum loading ($\mu_{Pt}$), and the carbon loading ($\mu_{C}$) [[40], [41], [42]]. The volume fraction of the Pt/C particles, ionomer phase, and pore phase are given by the following expressions:

(1) $$\epsilon_{Pt/C}=\frac{V_{Pt/C}}{V_{CL}}=\frac{1}{\gamma_{CL}}\left(\frac{\mu_{Pt}}{\rho_{Pt}}-\frac{\mu_{C}}{\rho_{C}}\right)$$

(2) $$\epsilon_{ion}=\frac{V_{ion}}{V_{CL}}=\epsilon_{Pt/C}\left(\frac{r_{\text{agg}}^{3}.Y_{i,agg}-r_{\text{agg}+\gamma_{i}}^{3}-r_{\text{agg}}^{3}}{r_{\text{agg}}^{3}\;\left(1-Y_{i,agg}\right)}\right)$$

(3) $$\epsilon_{P}=\frac{V_{P}}{V_{CL}}=1-\epsilon_{Pt/C}-\epsilon_{ion}$$

In the CL, there are two parts to the ionomer phase: one is inside the agglomerate and the other is the ionomer film.

The number of agglomerate particles per catalyst layer volume is given by:

(4) $$N=\frac{3\epsilon_{Pt/C}}{4\pi R_{\text{agg}}^{3}\left(1-Y_{i,agg}\right)}$$

The effective agglomerate surface area, meanwhile, is obtained as follows:

(5) $$A_{i,agg}=4\pi N{\left(R_{agg}+\gamma_{i}\right)}^{2}\epsilon_{P}$$

Two processes for delivering the reactants to the catalyst sites are described in the agglomerate model. The following electrochemical reaction occurs when oxygen in the pore spaces between the agglomerates in the cathode's catalyst layer diffuses through the covering ionomer film and penetrates the agglomerates to reach active sites before reacting with electrons and protons to form water.

$$O_{2}+4H^{+}+4e^{-}\;\to \;{2H}_{2}O$$

The electrochemical reaction rate is calculated as [4,43,44]:

(6) $$j_{C}=j_{0,C}.\left(1-\epsilon_{P}\right).\left(1-\varphi_{PtO}\right){.E}_{r}{.S}_{a}.\gamma_{CL}.n_{C}^{e^{-}}{\left(\frac{C_{O_{2}}^{\text{agg}}}{C_{O_{2}}^{\text{ref}}}\right)}^{\gamma_{c}}.\exp\left(-\frac{\alpha_{C}.F}{\text{RT}}.\eta_{act}\right)\exp\left(-\frac{\xi {.\varphi }_{PtO}}{RT}\right)$$

(7) $$j_{0,C}=j_{0,ref}\;\exp\left(-\frac{E_{c}}{R}.\left(\frac{1}{T}-\frac{1}{353.15}\right)\right)$$

Where $\alpha_{C}\text{,}$ ξ, $\gamma_{c}\text{,}$ and $\eta_{act}$ are the cathode transfer coefficient, the energy parameter for the Temkin isotherm, the oxygen reaction order, and the cathode overpotential, respectively. The oxide coverage $\varphi_{PtO}$ is a function of the cathode's potential U (cell voltage) as described in Ref. [45].

(8) $$\varphi_{PtO}=\frac{1}{\left(1+\exp\left(22.4\left(0.818-U\right)\right)\right)}$$

The effectiveness factor of the spherical agglomerate is given as:

(9) $$E_{r}=\frac{1}{\varphi }.\left(\frac{1}{\tanh\;\left(3\varphi \right)}-\frac{1}{3\varphi }\right)$$

Where the Thiele's modulus for the chemical reactions is defined as:

(10) $$\varphi =R_{agg}\;.\sqrt{\frac{j_{0,C}{.S}_{a}}{2F.C_{O_{2}}^{\text{ref}}.D_{O_{2}}^{\text{agg}}}.\exp\left(-\frac{\alpha_{C}.F}{\text{RT}}.\eta_{act}\right)}$$

### 2.3 Governing equations

Based on the agglomerate model developed in this work and the abovementioned assumptions, the fluid flow in the channels, GDLs, MPLs, and CLs was described through the mass and momentum conservation equations.

At any point in the channels, GDLs, MPLs, and CLs, the total mass and momentum conservations are can be summarized as below [46,47]:

(11) $$\rho \epsilon_{P}\nabla .\left(\vec{V}\right)=S_{mass}$$

(12) $$\rho \epsilon_{P}\nabla .\left(\vec{V}\vec{V}\right)=\epsilon_{P}\nabla .\left(\mu \nabla \vec{V}\right)-\epsilon_{P}\nabla P+S_{mom}$$

Where ρ is the density of the fluid mixture, $\epsilon_{P}$ is the porosity (it equals 1 in the flow channels), and $\vec{V}$ is the superficial velocity. $S_{mass}$ represents the source term for the mass equation presented in Table 1, and it exists only in the catalyst layers. P is the mixture's pressure, while μ is its dynamic viscosity. $S_{mom}$ denotes the source term for the momentum equation presented in Table 1. The density of the fluid mixture can be calculated using the ideal gas law, which is defined in Refs. [48,49] as $\rho =\frac{PM}{RT}<!--Q3:Table\;2\;\text{was}/\text{were}\;\text{not}\;\text{cited}\;\text{in}\;\text{the}\;\text{text}.\;\text{Please}\;\text{check}\;\text{that}\;\text{the}\;\text{citation}\left(s\right)\;\text{suggested}\;\text{are}\;\text{in}\;\text{the}\;\text{appropriate}\;\text{place},\text{and}\;\text{correct}\;\text{if}\;\text{necessary}.-->$ (see Table 2).

**Table 1:** Source terms in the governing equation.

Source term	Unit
$S_{mass}$ = $S_{H_{2}}$ + $S_{O_{2}}$ + $S_{H_{2}O}$ $S_{H_{2}}$ = $\left\{\begin{array}{c} -\frac{j_{a}}{2F}M_{H_{2}}\;\left(ACL\right)\\ 0\;\left(Other\;zones\right) \end{array}\right\}$ $S_{O_{2}}$ = $\left\{\begin{array}{c} -\frac{j_{c}}{4F}M_{O_{2}}\;\left(CCL\right)\\ 0\;\left(Other\;zones\right) \end{array}\right\}$ $S_{H_{2}O}$ = $\left\{\begin{array}{c} \frac{j_{c}}{4F}M_{H_{2}O}\;\left(CCL\right)\\ 0\;\left(Other\;zones\right) \end{array}\right\}$ $S_{mom}$ = $\left\{\begin{array}{c} -\frac{\mu }{K_{P}}\vec{V}\;\left(GDLs\;and\;CLs\right)\\ 0\;\left(Other\;zones\right) \end{array}\right\}$ $S_{T}$ = $\left\{\begin{array}{c} j_{a}\eta_{a}-\;\frac{T\Delta S_{a}}{2F}j_{a}+\sigma_{eff,m}\nabla \emptyset_{m}^{2}+\sigma_{eff,s}\nabla \emptyset_{s}^{2}+S_{l}\Delta h_{\mathrm{lg}}\;\left(ACL\right)\\ j_{c}\eta_{c}-\;\frac{T\Delta S_{c}}{2F}j_{c}+\sigma_{eff,m}\nabla \emptyset_{m}^{2}+\sigma_{eff,s}\nabla \emptyset_{s}^{2}+S_{l}\Delta h_{\mathrm{lg}}\;\left(CCL\right)\\ \sigma_{eff,m}\nabla \emptyset_{m}^{2}\;\left(MEM\right)\\ \sigma_{eff,s}\nabla \emptyset_{s}^{2}+S_{l}\Delta h_{\mathrm{lg}}\;\left(GDLs\right)\\ \sigma_{eff,s}\nabla \emptyset_{s}^{2}\;\left(CCs\right) \end{array}\right\}$ $S_{\alpha }$ = $\left\{\begin{array}{c} S_{H_{2}}\\ S_{O_{2}}\\ S_{H_{2}O} \end{array}\right\}$	Kg/$m^{3}.S$ Kg/$m^{2}.S^{2}$ W/$m^{3}$ Kg/$m^{3}.S$

Where M is the molar mass of the fluid, T is the temperature, and R is the ideal gas constant.

Darcy's law was used to describe flow through porous media, and this is expressed as [47,50]:

(13) $$\nabla P=-\frac{\mu }{K_{P}}\left(\epsilon_{P}\vec{V}\right)$$

Where $K_{P}$ is the permeability of the fluid flow into porous media.

Due to the existence of multiple fluid species ($H_{2},O_{2},N_{2},and\;H_{2}O)$, the law of Fick was used to describe the fluid diffusion in porous electrodes. The general species conservation equation for the fluid mixture can be given by a Maxwell–Stefan type equation [46,50]:

(14) $$\rho \epsilon_{P}\frac{\partial }{\partial t}\left(C^{\alpha }\right)+\rho \nabla .\left(\vec{V}C^{\alpha }\right)=\rho \epsilon_{P}\nabla .\left(\sum D_{j}^{\alpha }\nabla C^{\alpha }\right)+S_{\alpha }$$

Where C is the concentration of the species, D is the diffusion constant, and $S_{\alpha }$ represents the source term for the species transport conservation equation presented in Table 1.

In this work, the PEMFC functions regularly, the reactant fluid mixtures are ideal fluids, the flow in the channels is laminar, and the GDLs and CLs are homogenous and isotropic. Thus, the gaseous species conservation equation in the channels, GDLs, MPLs, and CLs and the energy conservation equation can be given as, respectively:

(15) $$\rho \nabla .\left(\vec{V}C^{\alpha }\right)=\rho \epsilon_{P}\nabla .\left(D^{\alpha }\nabla C^{\alpha }\right)+S_{\alpha }$$

(16) $$\rho \nabla .\left(C_{P}\vec{V}T\right)=\nabla .\left(K_{eff}\nabla T\right)+S_{T}$$

Where $C_{P}$ is the specific heat, $K_{eff}$ is the effective thermal conductivity, and $S_{T}$ is the thermal source term that is presented in Table 1 [51,52].

### 2.4 Assumptions for boundary conditions

Boundary conditions must be supplied in order to complete the model description. Considering the fully developed laminar flow, the velocity is defined at the inlet of the gas channels, and this is calculated based on a flat active surface area of CL $A_{XZCL}$, the cross-sectional inlet area of the flow channel $A_{C,inlet}\text{,}$ the reference current density $I_{ref}\text{,}$ the inlet molar concentration C, the stoichiometric ratio σ, and the Faraday constant F.

At the anode and cathode channel inlet, the velocity is given by:

(17) $$v_{inlet,a}=\frac{\sigma_{a}.I_{ref}.A_{XZCL}}{2FA_{C,inlet}.C_{H_{2}}}$$

(18) $$v_{inlet,c}=\frac{\sigma_{c}.I_{ref}.A_{XZCL}}{4FA_{C,inlet}.C_{O_{2}}}$$

The mass flow rate m is given as a function of the operating current density I, the stoichiometric σ, the MEA area $A_{act}$, and the species concentration C.

At the anode and cathode channel inlet, the mass flow rate is therefore specified as [53]:

(19) $$m_{a}=\frac{\rho_{a}.I.{\sigma_{a}.A}_{act}}{2FC_{H_{2}}}$$

(20) $$m_{c}=\frac{\rho_{c}.I.{\sigma_{c}.A}_{act}}{2FC_{O_{2}}}$$

The main overall assumptions of this proposed model are as follows.

a) The PEMFC operates at a steady-state condition;
b) The reactants are ideal gases;
c) The GDLs, MPLs, and CLs are homogeneous and isotropic;
d) The reactant gases cannot diffuse across the membrane;
e) The gas flow in the fuel cell is laminar;
f) The effect of gravity is negligible.

## 3 Validating the model

**Fig. 3:**
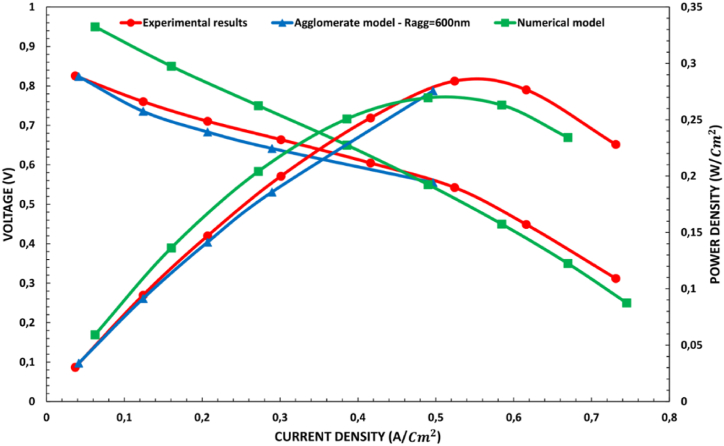
Comparison of the agglomerate and numerical models with the experimental results.

To validate the accuracy of our proposed model, we compared the results of this study with the experimental results reported by Yan et al. [54], as shown in Fig. 3. The 25 ${\text{Cm}}^{2}$ PEMFC used in the experiment was monitored under a working temperature, pressure, and relatively humidity of 353.15 K, 1 atm, and 100 %, respectively, while the platinum loading was $0.4\;mg/{\text{Cm}}^{2}$ for the anode and cathode CLs. By comparing the polarization curves in Fig. 3, we can see that there is a good level of precision, with the agglomerate model and 3D CFD model reasonably predicting the performance of PEMFC. Note that values of $R_{agg}=600nm$ and $Y_{i,agg}=0.2$ were adopted in the agglomerate model. To conduct the 3D CFD model, following the geometry step, we proceeded with meshing using a relatively small number of elements, approximately 500k elements of the highest quality. The simulation successfully converges within 3 h with standard initialization and steady state.

## 4 Results and discussion

### 4.1 Effect of the agglomerate parameters on cell performance

Having developed and validated our proposed model of a CL cathode based on the agglomerate model and the Pt-oxide-coverage-dependent kinetics equation for ORR, we then investigated the effect of changing the agglomerate parameters on cell performance. Thus, $R_{agg}$ was varied from 100nm to 900nm with $Y_{i,agg}$ being fixed at 0.6, while $Y_{i,agg}$ was varied from 0.3 to 0.6 with $R_{agg}$ being fixed at 100nm.

**Fig. 4:**
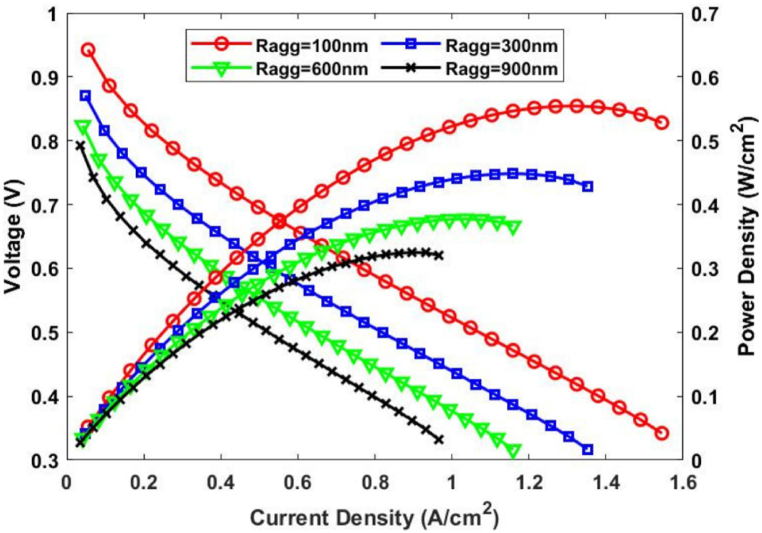
Effect of varying $R_{agg}$ on cell performance in terms of the a) polarization and b) power density curves.

**Fig. 5:**
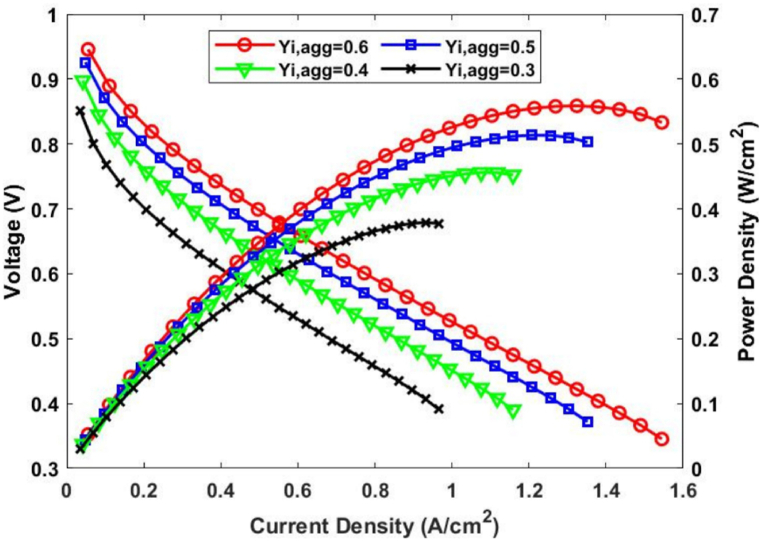
Effect of varying $Y_{i,agg}$ on cell performance in terms of the a) polarization b) and power density curves.

The cell's performance was expressed and evaluated in terms of the polarization and power density curves. As can be seen in Fig. 4, varying $R_{agg}$ has a significant impact on the cell's performance at medium and high current densities, with the maximum power density reaching $0.554\;W/{\text{Cm}}^{2}$ when $R_{agg}$ is set at 100nm, while the minimum power density value of $0.324\;W/{\text{Cm}}^{2}$ occurs with an $R_{agg}$ fixed at 900nm. The impact of varying $Y_{i,agg}$ on cell performance is illustrated in Fig. 5. At medium and high current densities, $Y_{i,agg}$ also clearly influences the current density, with the power density decreasing from $0.558\;W/{\text{Cm}}^{2}$ to $0.379\;W/{\text{Cm}}^{2}$ as the $Y_{i,agg}$ is decreased from 0.6 to 0.3.

**Fig. 6:**
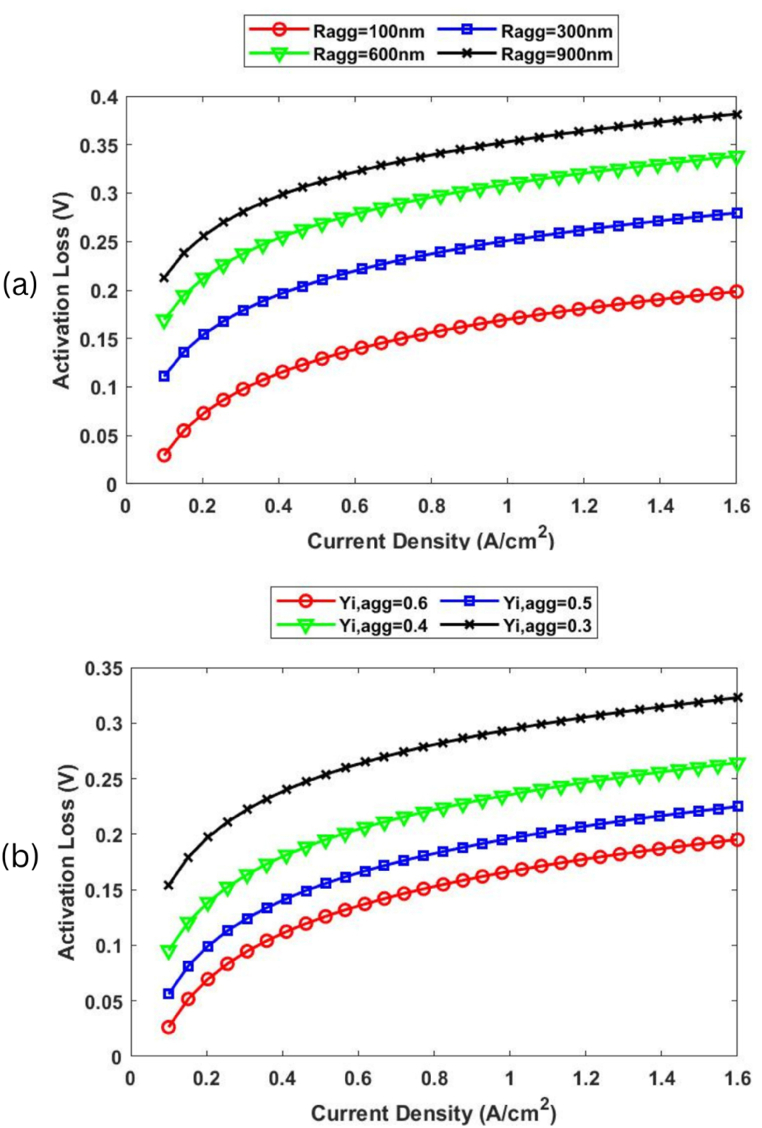
The effect of varying a) $R_{agg}$ and b) $Y_{i,agg}$ values on activation loss.

**Fig. 7:**
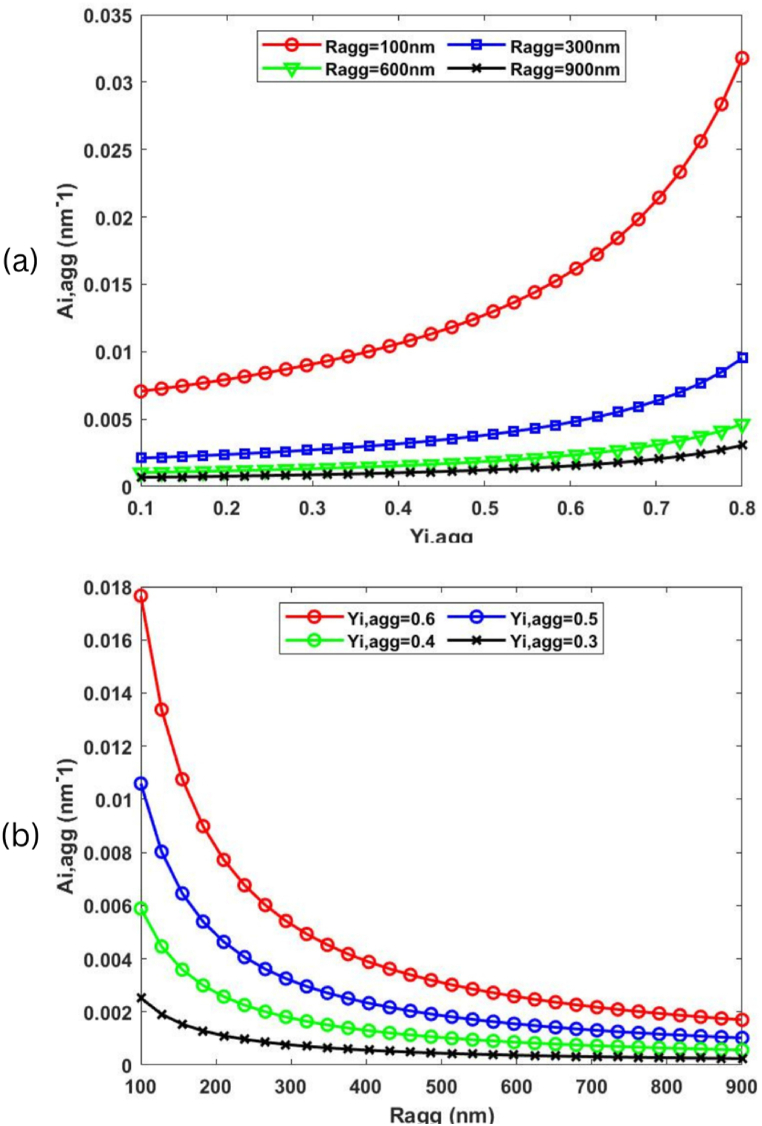
The effect of varying a) $R_{agg}$ and b) $Y_{i,agg}$ values on the effective agglomerate surface area.

Fig. 6a presents the activation loss for the different $R_{agg}$ values (100, 300, 600, and 900nm) when $Y_{i,agg}$ is fixed at 0.6, while Fig. 6b shows how activation loss is also very sensitive to varying $Y_{i,agg}$ values (0.3, 0.4, 0.5 and 0.6) when $R_{agg}$ is fixed at 100 nm. Next, Fig. 7 shows how the effective agglomerate surface area is also strongly influenced by variations in the $R_{agg}$ and $Y_{i,agg}$ values.

### 4.2 Effect of varying output voltage on fuel cell performance

For this part of the study, we considered a typical hydrogen–oxygen PEMFC, as shown in Fig. 1, with the detailed geometric parameters listed in Table 3. The operational temperature and pressure were 353.15K and 1 atm, respectively. The CFD software ANSYS FLUENT was used for simulations here with the aim of studying and analyzing the temperature distribution, the hydrogen and oxygen mass fractions, and the concentration of hydrogen and oxygen inside the anode and cathode. In addition, we sought to analyze the influence of output voltage on the concentration of gases, mass fraction, temperature distribution, and ultimately the performance of the PEM fuel cell.

**Table 2:** PEMFC operating conditions.

Parameter	Anode	Cathode
Reactant gas	Hydrogen	Oxygen
Operating pressure	1atm	1atm
Operating temperature	353.15 K	353.15 K
Relative humidity	100 %	100 %
Stoichiometric ratio	1.5	2.0

**Table 3:** Geometric parameters and materials properties for simulation.

Parameter	Value
BP length/height/width (mm)	30/2/2
Flow channel length/height/width (mm)	30/1/1
Thickness of GDL/CL/MEM (mm)	0.2/0.15/0.2
Porosity of GDL/CL	0.6/0.2
Absolute permeability of GDL/CL/MEM/($m^{2})$	3. $10^{-12}$/2. $10^{-13}$/1. $10^{-18}$

**Fig. 8:**
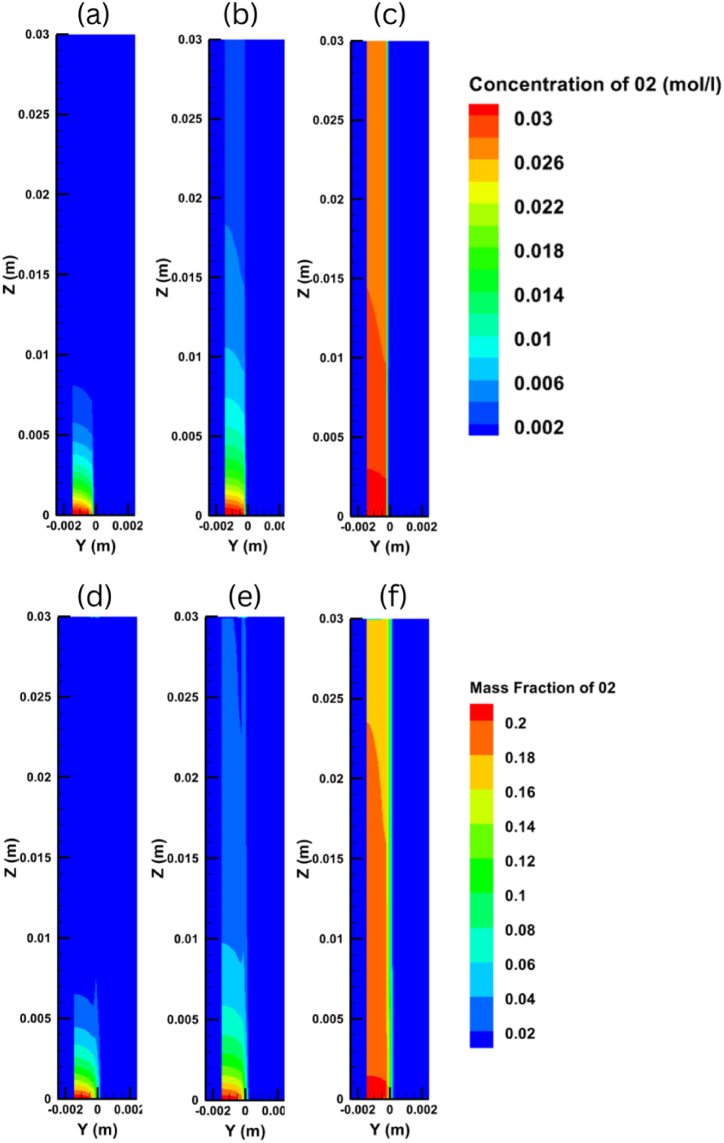
Oxygen concentration distribution at different voltages of (a) 0.35V, (b) 0.65V, (c) 0.95V and Oxygen mass fraction distribution at different voltages of (d) 0.35V, (e) 0.65V, (f) 0.95V on the y-z plane.

**Fig. 9:**
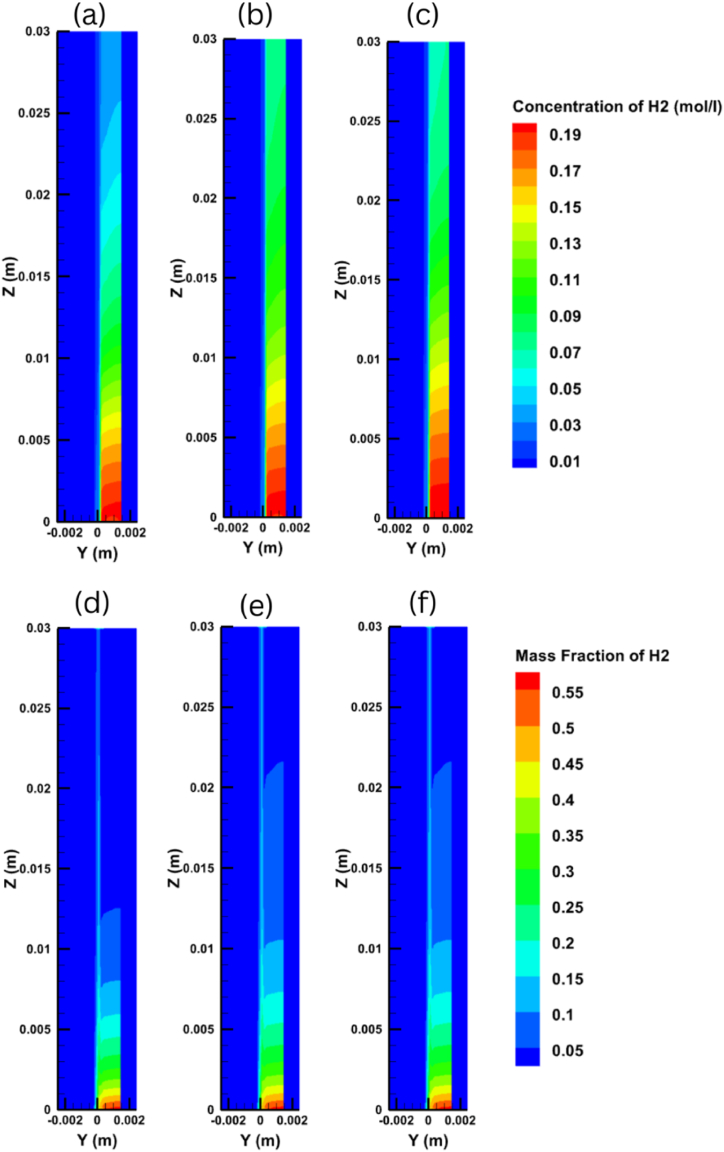
Hydrogen concentration distribution at different voltages of (a) 0.35V, (b) 0.65V, (c) 0.95V and Hydrogen mass fraction distribution at different voltages of (d) 0.35V, (e) 0.65V, (f) 0.95V on the y-z plane.

When seeking to improve the performance of a PEM fuel cell, it is very important to understand the hydrogen and oxygen transport inside the cell and along the anode and cathode sides. Fig. 8 shows the variations in oxygen concentration and mass fraction distribution on the y-z plane for output voltages of 0.35 V, 0.65 V, and 0.95 V, while Fig. 9 does the same for the hydrogen concentration and mass fraction distribution. It is clear from these figures that the concentration and mass fraction of the gases decrease along the flow channel due to them being consumed inside the cell. It can also be discerned that decreasing the output voltage can accelerate the electrochemical reaction process, which in turn speeds up the rate that the gases are consumed in the fuel cell.

**Fig. 10:**
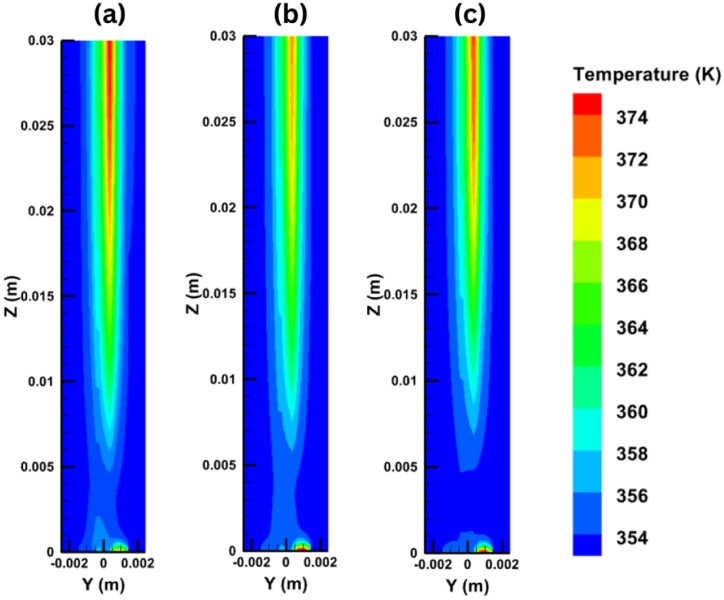
Temperature distribution on the y-z plane at different voltages of (a) 0.35V, (b) 0.65V, (c) 0.95V.

**Fig. 11:**
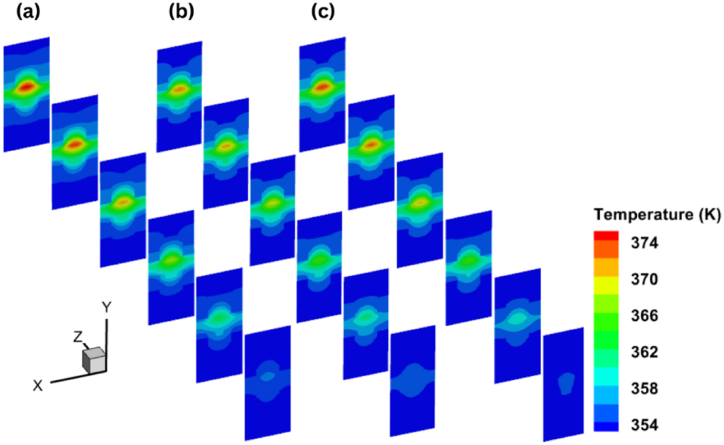
Temperature distribution for six x-y planes at different voltages of (a) 0.35V, (b) 0.65V, (c) 0.95V.

Next, Fig. 10 shows the temperature distribution on the y-z plane inside the PEM fuel cell under different output voltages. It can be clearly seen that the temperature is high at the outlet region and very low at the inlet region, and it is higher at the cathode side that it is at the anode side due to the reversible and irreversible entropy production. Fig. 11 presents the temperature distribution for six x-y planes along the PEM fuel cell, from the inlet to the outlet of the cell.

## 5 Conclusion

An agglomerate model based on agglomerating the catalyst particles and the Pt-oxide-coverage-dependent kinetics equation for ORR was developed and applied to characterize the electrochemical kinetics caused by the sluggish ORR in the CL cathode and investigate the impact of varying some of the key agglomerate parameters ($R_{agg}$ and $Y_{i,agg}$). In addition, a numerical model was developed using CFD methods and the ANSYS FLUENT software. The primary goal of this study was to compare the agglomerate and numerical models with some experimental results to verify their accuracy. In addition, the geometry of the computational domain and the governing equations at any point in the PEMFC's channels in three-dimensions have been given in this work. The main findings of this study can be summarized as follows:

• Varying $R_{agg}$ and $Y_{i,agg}$ affects cell performance at medium and high current densities.
• When $R_{agg}$ is decreased and $Y_{i,agg}$ is increased, the current density is significantly enhanced at medium and high currents.
• Activation loss increases as $R_{agg}$ increases and as $Y_{i,agg}$ decreases.
• The effective agglomerate surface area increases as $R_{agg}$ decreases and $Y_{i,agg}$ increases.
• A decrease in the output voltage can accelerate the electrochemical reaction process.
• The temperature is higher at the cathode side than at the anode side due to the reversible and irreversible entropy production within the cell.

## Data availability statement

No additional data are available to report for this manuscript.

## CRediT authorship contribution statement

**Abdelaziz Samris:** Writing – original draft, Visualization, Validation, Methodology, Investigation, Formal analysis, Conceptualization. **Hamid Mounir:** Writing – review & editing, Supervision, Software.

## Declaration of competing interest

The authors declare that they have no known competing financial interests or personal relationships that could have appeared to influence the work reported in this paper.

## List of Symbols

$R_{agg}$: Agglomerate Radius (m)
$Y_{i,\text{agg}}$: Volume fraction of ionomer within the agglomerate
$\epsilon_{\text{Pt}/C}$: Volume fraction of Pt/C particles in CL.
$\epsilon_{\text{ion}}$: Volume fraction of ionomer in CL.
$\epsilon_{P}$: Volume fraction of CL porosity
$\mu_{C}$: Carbon loading (mg. ${cm}^{-2}$)
$\mu_{\text{Pt}}$: Platinum loading (mg. ${cm}^{-2}$)
$\rho_{C}$: Density of carbon (Kg. ${cm}^{-3}$)
$\rho_{\text{Pt}}$: Density of platinum (Kg. ${cm}^{-3}$)
$\gamma_{CL}$: Catalyst layer thickness (m)
$\gamma_{i}$: Thickness of ionomer film (m)
$j_{0,C}$: Cathode exchange current density of oxygen reduction reaction (A/ $m^{2}$)
$j_{0,ref}$: Cathode reference exchange current density of oxygen reduction reaction (A/ $m^{2}$)
$\varphi_{PtO}$: Platinum oxide coverage
$\alpha_{C}$: Cathode transfer coefficient
F: Faraday constant
R: Ideal-gas constant
T: Temperature (K)
$C_{O_{2}}^{\text{ref}}$: Reference concentration of oxygen (mol/ $m^{3}$)
$C_{O_{2}}^{\text{agg}}$: Concentration of oxygen in the cathode reaction layer (mol/ $m^{3}$)
$D_{O_{2}}^{\text{agg}}$: Diffusion coefficient of hydrogen in agglomerates ($m^{2}$/ $S^{-1}$)
$n_{C}^{e^{-}}$ =: Number of charges for the cathode
ξ: Energy parameter for Temkin isotherm (J/mol)
$\gamma_{c}$: Oxygen reaction order
η: Over potential (V)
$E_{c}$: Activation energy in oxygen reduction reaction (J/mol)
$S_{a}$: Specific surface area per unit volume in each agglomerate particle ($m^{-1}$)
μ: Dynamic viscosity (Pa.S)
$K_{P}$: Permeability ($m^{2}$)
$\Delta S_{a}$: Entropy of hydrogen oxidation (J/mol.K)
$\Delta S_{c}$: Entropy of oxygen reduction (J/mol.K)
S: Liquid water saturation
σ: Electron/Proton conductivity ((S/m), Surface tension (N/ $m^{2}$)
∅: Potential (V)
$\Delta h_{\mathrm{lg}}$: Latent heat of condensation/evaporation (J/mol.K)
